# Intelligence

**Published:** 1829-04

**Authors:** 


### INTELLIGENCE.

### MONTHLY REPORT OF PREVALENT DISEASES.

Hooping cough, scarlet fever, and bronchial inflammation, have been very prevalent, and more than commonly severe, amongst children. Measles have also been frequent.

Two cases of hooping-cough have proved fatal within our knowledge. In each the bowels were obstinately constipated, notwithstanding the free use of purgative remedies, and in each symptoms of great cerebral disturbance suddenly occurred. The infants soon became comatose, and died. Upon examination after death, the ventricles of the brain were found to contain, in one case, about an ounce, and in the other an ounce and a half, of fluid. Several instances of mild scarlatina have been followed by anasarca of the extremities and considerable general debility. Mild purgatives, diuretics, and light tonics quickly relieved these patients.

Although we have had abundant opportunities of seeing rheumatism in all its forms, we do not remember a case similar to the following:

A healthy man, a farrier by trade, after exposure to cold, was attacked with slight rheumatic fever. His arms at first suffered, and very severely. The pains and superficial inflammation then left the arms, and settled in the legs. The left testicle then became very painful: it was considerably enlarged, and very hot to the touch; the surface of the scrotum smooth and red. The patient was now free from pain in every other part. He had hitherto considered the attack to be too trifling to require medical treatment, but he now applied for our advice. He was directed to remain quiet, to apply leeches to the scrotum, and afterwards tepid lotions. The bowels were freely opened. In two days the testicle was no longer painful, and but slightly enlarged. The appearance of the scrotum was nearly natural. He now suffered much from pain in the ankles and forearms, both of which parts were swollen and inflamed. Tepid lotions of spirits of wine and camphor mixture were directed to be constantly applied to the affected parts. The Vinum Sem. Colchici was given three times a day, in doses of thirty minims, and an anodyne at bedtime. In three days he was perfectly well, and has since remained free from any other complaint than a slight tenderness of the testicle. He had not had gonorrhœa for several years.

*College of Physicians.*—The second meeting of the season took place on Monday evening, and was numerously attended. A paper by Dr. HOLLAND, entitled "Remarks on the Use of Sudorific Medicines," was read. The object of this paper is to draw attention to the principles upon which internal sudorific remedies are employed, and to suggest some observations calculated to render more explicit our views as to their nature and operation. After remarking on the great uncertainty of effect attending the use of this class of remedies, even in cases where natural sweating is a symptom habitually present in the progress of the disease, and upon the difficulty of discriminating as to the effects of different medicines of the class, Dr. H. proceeds to examine the principles upon which the use of sudorifics has chiefly been founded. A principal one appears to be the fact of the suspension of various morbid actions in sequel to natural sweating, as witnessed in the simple paroxysms of fever, in many of the phlegmasiæ, &c. Though this analogy is plausible, however, yet it is open to many doubts. Various considerations are stated by the author, justifying these doubts; and he infers that, in the present state of our knowledge, it is more correct in principle, and safer in practice, to consider the sweating stage, or critical sweat, as one of the series of changes constituting fever, than as the efficient cause of the subsidence of the paroxysm; rather as an index of the changes occurring in the vascular system, than as the agent in producing such changes. In reference to the same argument, he recites some of the many instances of natural perspiration, under states of disease, without relief, or often with aggravation of danger to the patient.

In the succeeding part of the paper, Dr. H. considers particular sudorific remedies upon the general principles before laid down; doubting altogether the efficacy of many medicines so classed, and attributing the probable influences of others, as of the antimonials, chiefly to their effect upon the circulation, and in diminishing febrile action. He considers their employment in practice will generally be more successful in proportion as this principle is kept in view. He expresses his concurrence with the opinion of FREIND as to the superior advantages of opium over most other diaphoretics; and makes some remarks on the diminished and less effectual use of opiates in modern practice.

After some further observations on bloodletting, diluents, &c. as affecting the action of the capillaries of the skin, Dr. H. concludes his paper by the following general deductions as to the use of sudorifics: 1st. That it is for the most part more expedient in practice to have regard to the changes in the vascular system producing diaphoresis, than to the action of sweating itself; 2d, that the amount of perspiration is seldom a measure of the benefit obtained; and 3dly, that to make this a criterion, or primary object, is likely often to give a wrong bias to the treatment of disease.

*Climate of the Azores.*—The learned President read a letter from a correspondent, in which he spoke very favorably of the climate of the Azores for invalids, especially those labouring under consumption, a complaint which he stated to be very uncommon in these islands. The maximum temperature during the year is about eighty-four, the minimum about forty-eight, of Fahrenheit. The writer gave an account of some hot springs, from which baths are made, enjoying high repute; and of various mineral waters, some of which are chalybeate, others contain a large quantity of free carbonic acid, and yet others are merely saline.

*A Compendium of the Introductory Lecture of* J. G. GUTHRIE, Esq. F.R.S. *delivered before the Royal College of Surgeons, the* 11*th of March*, 1829.

A considerable time before the commencement of the Lecture, the theatre was crowded to excess, and many members were unable to obtain admission. Precisely at four o'clock, the College dignitaries, the council, and the select visitors, presented themselves, and occupied the exclusive seats. The Professor immediately followed, and expressed himself to the following effect:

In accepting the honour which the council have conferred upon me, by appointing me their Professor of Anatomy and Surgery, I have to express my grateful feelings for their kindness, and to beg their indulgence towards the efforts I am about to make. I am fully aware of the magnitude and importance of the office which I undertake; an office which implies the imparting of knowledge to so learned a community as that I now address. The council, aware of the constant, though gradual, progression of anatomical and chirurgical knowledge, as well as of the proneness of individuals to adopt and pertinaciously adhere to favorite theories and speculations, have formed the wise regulation of appointing their professors for limited periods. In lectures directed to a body of men so distinguished for originality of conception and soundness of judgment as the Royal College of Surgeons, it is not enough to describe the processes of bones, the origins and insertions of muscles, the course and relative position of blood-vessels, nerves, and absorbents: these are the mere elementary parts of the science. A novelty of doctrine is necessary to fix the attention, to rouse into action the speculative powers of original minds, by presenting a perpetual series of new experiments, and consequent inferences. If professors were appointed for life, these results would not accrue: the limited extent of the human faculties, in a single individual, could, for so long a period, afford little more than an exposition of principles already demonstrated, and universally acknowledged. I need scarcely say, gentlemen, that such a mode of proceeding could not be suffered by this enlightened College.

The numerous truths which have of late years been elicited in anatomy and surgery, give an appearance of fallacy to their principles, which the exact sciences are not subject to: the seemingly well-established axioms of one age become, through the accumulation of facts, merged in the still more general laws of the next, and these again become involved in future generalizations. I ascribe my own appointment to this chair, in part to my standing in the College, and in part to the partiality of the council. I do not shrink from the responsibility incurred by the acceptance of this office; and I shall endeavour to inculcate on your minds the importance of the study of human anatomy and surgery, which will form the subjects of my future lectures. The progress which these sciences have hitherto made is ascribable to the aggregation of knowledge; the force of individual exertion is feeble indeed; and when considered in relation to the vast domain of nature, is calculated to teach a lesson of humility, to convey an impressive idea of the nothingness of man, compared with the universal development of the supreme Power. Some rare examples of sublime genius have from time to time appeared, and have earned for themselves an immortality of fame, whose achievements in science throw into the shade the aggregate transactions of an age. The rare genius and uncommon industry of John Hunter projected and accomplished the formation of a museum, which is now an ornament to the walls of this College, and a noble monument of its illustrious author. I

am happy it is the determination of the council to follow in the track of this great man, and to devote a portion of the funds which have for years been silently accumulating, to the cultivation and diffusion of anatomical and surgical knowledge.

The structure of man is perfect: nothing can be added to, or altered in, the constitution of his frame, without deterioration; nothing can be taken away, where there is no superfluity. Such is the beautiful mechanism of the human body, that a closer study of its various movements would contribute to the improvement of mechanics in general. In man are contained the rudiments of all organs of other animals. Those organs which in man are small, and hardly developed, are in some animals more perfect, and perform an evident function; and organs which can scarcely be detected in one class of animals perform an important part in the functions of another: thus it is that the comparative examination of the subjects of the animal kingdom tends to illustrate the functions of the whole.

Any observations in explanation of the influence of the knowledge of anatomy, or physiology and pathology, would justly be considered trite. With respect to the infliction of experiments on minor animals, for the purposes of physiological investigation, I do not see any great objection, provided such experiments are performed with determinate views and a rational purpose. It ill becomes those who destroy thousands of those animals which they have for months nurtured and caressed, for the purpose of ministering to the grosser appetites, to carp at physiological experimentalists, whose efforts are directed to the noblest ends. I have myself never performed any experiments on living animals; I have always been influenced by the feeling so finely expressed by the poet.

Those experiments which I have directed to be performed were indispensable to the acquisition of certain facts, and conducted with the greatest possible humanity.

The Lecturer, who had hitherto read his communication, now closed his book, and thus addressed the president:

Sir,—I beg to address you, as Professor of Surgery to this College; as professor of an art which does not hold so high a rank in society as it merits, or so high as that it attained amongst the ancients. For its early history I shall refer you to the song of that immortal bard and poet, with whom we are made familiar even in boyhood; that sublime poet, whose influence, after a lapse of some thousand years, of late induced Europe not only to draw the sword, but (what is more extraordinary) to draw the purse-strings, in favor of his unhappy country Greece. It is Homer who relates that, when a pestilence appeared in the Grecian army before Troy, the infected persons were directed to apply to the priest of Apollo. But when Machaon, one of the chiefs of the army, and himself a surgeon, was wounded, he was taken to the camp, that he might be attended by those skilled in dressing wounds. The anxiety entertained in the public mind respecting him was not occasioned so much by his rank as a prince, or his talents as a leader, as by his skill as a surgeon. In those days surgery was practised by princes and philosophers.

I beg to refer you also to the Augustan age, when a surgeon was appointed to each legion, and styled Medicus Vulnerarius. These officers were held in high honour: they were exempted from taxes, made citizens of Rome (a distinction coveted by, and often refused to, crowned heads,) and distinguished by gold rings, as having the rank of Equites. It is evident from these facts that the distinction between medicine and surgery did not formerly exist. In the time of Hippocrates, 400 years before Christ, the three branches were frequently practised by one individual. In the fourth century, the irruption of the Goths and Vandals involved in the general waste all vestiges of the art of surgery in the western world; and the destruction of the Alexandrian library by the Saracens completed its fall in the east. In Europe during the dark ages the practice of medicine and surgery was confined to ecclesiastics. The surgical part was most frequently performed by their servants. The priests were prevented by the canons of their church from practising surgery, and superstition amongst the Mahomedans led to a similar degradation of the art in that community. The wars of the Saracens, however, soon taught them the necessity of cultivating surgery; and we accordingly find that, while Europe was involved in intellectual darkness, many bright names flourished amongst the Arabians. In Christendom, where war likewise gave an impetus to the study, a distinction arose amongst the medical men of Ministri Honorati, who performed the part of physicians, and the Ministri Serviles, or barber surgeons. At a council held at Tours in 1163, it was declared improper for an ecclesiastic to perform any operation on the naked human body; and two bulls were issued by two several popes in the eleventh century, to a similar purpose. At this period surgery was certainly at its lowest ebb.

In 1168, Jean Pitard, who may justly be considered the greatest benefactor to surgery in modern times, was the first to raise the art from its degraded condition. Although of humble birth, he raised himself to the rank of personal surgeon to St. Louis, whom he accompanied in his disastrous crusades, and whose friend and counsellor he afterwards became. On his return from Egypt with his master, he employed the influence which he possessed over the royal mind, not in contributing to his own aggrandizement, but in raising the character of the profession. He induced the king to establish an academy of surgery at Paris, and to appoint professors, who were invested with powers to examine all persons aspiring to the rank of surgeon.

William Vavasour, physician to Francis I., next to Pitard deserves our respect. By his interest with the king, he procured the elevation of the academy of surgery to the dignity of a university; and he himself was a most distinguished and scientific practitioner.

In 1615, the less eminent practitioners who were dispersed through the provincial towns, and could not, of necessity, be members of the university, conspired against that institution, and procured a royal edict for their association with it.

In 1731, a sacred band of surgeons offered a firm resistance to the degradation of the surgical profession, and succeeded in reestablishing the academy, and thus contributed to remedy the ills occasioned by the inundation of the barber surgeons.

I have not yet noticed the progress of surgery in this country. It is well known to those read in history, that during this period all physicians and surgeons were imported from the continent. As there were no schools on this island, our youths were obliged to resort to Paris, and other foreign academies. But, although there were no schools of surgery in this kingdom, many great surgeons flourished amongst us. Wiseman, serjeant chirurgeon to Charles II., is a writer whose work may be consulted with advantage even now; the great Cheselden was at twenty-two years of age a teacher, and at twenty-five published his celebrated work. So great was his reputation, that the French academy sent over Morand, their secretary, to observe and report upon his method of operating.

In 1745, the corpo:ation of surgeons was established, and have since that period been actively engaged in the cultivation of anatomy and surgery. No institution could do more for the advancement of the professional character than this has done; and, without a doubt, it will raise the name of surgeon to as great consideration as it has ever enjoyed, provided the barriers and trenches which protect its high offices are not removed. Should this unfortunately occur, it would take a century to repair the mischief that would ensue, and to restore the surgeon to his pristine respectability. Not that I would exclude any man *absolutely* from any situation in the College; but certain qualifications and conditions are necessary, to preserve the consideration of the world.

This consideration will not be obtained unless, like the professors of the law, the professors of physic and surgery show themselves competent to think and speak on other subjects besides those immediately connected with their profession. I know not what ban is set upon them to prevent it. I would have the medical profession represented in Parliament. We see that tradesmen of all classes have efficient representatives in the great council of the nation, who decide on all questions affecting the weal of the nation, and even feel themselves fully competent to determine upon the propriety and necessity of anatomical dissections; while not a single surgeon or physician, of all those whose wealth, abilities, and character qualify them for the task, is found to defend the interests of the medical body in that high assembly. From whence does this arise? Is all ambition withered within us? Are there not men among us with the same desires, the same views and energies, that urge forward the members of every other class or profession? or are our numbers, importance, and rank in society, not sufficient to entitle us to consideration, and to warrant the assertion of our claims? I trust our remaining stationary is not to be attributed, as we have been accused, to a desire of accumulating money; but that we can love honour, as much as wealth, and are as capable of exertions for the one as the other. I see around me many who have just completed their studies, and are entering upon the duties of the profession. I would anxiously urge them, not only to the active exercise of these duties, but also to the acquirement of scientific attainments in every branch of knowledge; for it is by such studies that the profession will be advanced in the estimation of the world; and, by labour thus directed, they may be enabled proudly to assert their right to rank, if not as the first, at least as the equal, of all other learned professions; and if ambition be necessary to urge them forward, let them ask themselves what bars their way to honours, more than that of any other profession? The answer is, Nothing; the road lies before you.

I beg I may not be misunderstood. It is not every man who has a few thousand pounds in his pocket that ought to think himself competent to stand forward as the champion of the profession. No: sixty winters must have shed its hoar on his locks who should attempt it. He must have long enjoyed the suffrages of that profession, and have acquired an independent fortune. Cannot one man be found in each of its branches of physic and surgery, who answers to this description. I can, sir, almost lay my hand upon them both.

I will not detain you, gentlemen, any longer on this subject; but if I have been happy enough, in the course of these observations, to raise the ambition, or spur on to more active exertions, a single individual, I shall rest satisfied that these introductory remarks have not been made in vain.

Throughout the above eloquent discourse, Mr. GUTHRIE evinces his characteristic ardour for the honour and advancement of the profession. He very judiciously urges all who are cultivating their professional studies to extend their inquiries to those other accomplishments which men of liberal education are expected to possess. The public in general must form an estimate of medical talent, of which they can rarely be competent judges, by proofs of knowledge upon subjects of which they are, or at least fancy they are, capable of forming an opinion. He who is really a zealous student need not fear to step beyond the pale of professional information. Occasional relaxation is necessary to every mind, and may always be obtained by a judicious alternation of study. The general character of the lectures which Mr. Guthrie has as yet delivered at the College may be conveyed in a few words: They prove him to possess a very acute and active mind. He conceives his subject clearly, and arranges it perspicuously. His language is correct and vigorous. In medical discussions, we have often listened to Mr. Guthrie with much pleasure as a public speaker; but it was yet to be seen how he would acquit himself before so large a body of his peers in the responsible character of Professor. He has certainly passed the ordeal with the highest credit to himself.

Mr. MAYO next takes the professorial chair, and Mr. Guthrie afterwards delivers a course of surgical lectures.

---

*New Regulations with regard to Medical Degrees.*

*Cambridge, March* 6.—The candidates for the degree of M.B., in addition to the examination of the Regius Professor of Physic, to be examined by the Professors of Anatomy, Chemistry, and Botany, each in his own science, previously to the performance of the public exercises in the schools; and that every candidate attend at least one course of lectures on each of the above subjects. He may offer himself for examination any time during his fifth year from admission, but not earlier. That no person be admitted to pass to the degree of Bachelor of Medicine, without having been admitted to any college, who after this date shall, during the time of his being in *statu pupillari*, have been engaged in the practice of pharmacy or midwifery, or in any trade whatever.

### METEOROLOGICAL JOURNAL,

By Messrs. HARRIS and Co. Mathematical Instrument Makers, 50, High Holborn.

| February | Rain gauge. | Moon. | Thermom. | | | Barometer. | | De Luc's Hygrom. | | Winds. | | Atmospheric Variations. | | |
|---|---|---|---|---|---|---|---|---|---|---|---|---|---|---|
| | | | 9 A.M. | MAX. | MIN. | 9 A.M. | 10 P.M. | 9 A.M. | 10 P.M. | 9 A.M. | 10 P.M. | 9 *a.m.* | 2 *p.m.* | 10 *p.m.* |
| 20 | | | 44 | 49 | 39 | 29.50 | 29.46 | 65 | 67 | WSW | SW | Foggy | Fine | Fine |
| 21 | | | 42 | 50 | 40 | .11 | .03 | 68 | 66 | SSE | S | Rain | Cloudy | Cloudy |
| 22 | .08 | | 42 | 46 | 37 | .14 | .25 | 66 | 65 | SE | SE | Cloudy | Sleet | Cloudy |
| 23 | | | 37 | 40 | 34 | .41 | .42 | 64 | 63 | E | E | Fine | Fine | Fine |
| 24 | | | 36 | 40 | 35 | .33 | .42 | 62 | 67 | E | ENE | Fine | Fine | Fine |
| 25 | | | 38 | 42 | 36 | .76 | .93 | 69 | 72 | E | E | Sl. Fog | Fine | Cloudy |
| 26 | | ☾ | 39 | 41 | 39 | .90 | .86 | 74 | 69 | E | ESE | Foggy | Rain | Rain |
| 27 | .04 | | 41 | 41 | 33 | .98 | 30.17 | 69 | 67 | ENE | ESE | Foggy | Show'ry | Fine |
| 28 | .02 | | 34 | 40 | 27 | 30.24 | .18 | 66 | 66 | SE | SE | Fine | Fine | Fine |
| Mar. | | | | | | | | | | | | | | |
| 1 | | | 32 | 38 | 32 | .12 | .04 | 65 | 65 | SE | ENE | — | — | — |
| 2 | | | 34 | 36 | 33 | .01 | .12 | 64 | 65 | ENE | NE | Foggy | — | Cloudy |
| 3 | | | 36 | 40 | 36 | .21 | .18 | 65 | 66 | ENE | NE | Sl. Fog | — | Cloudy |
| 4 | | | 40 | 42 | 33 | .02 | .05 | 68 | 65 | ENE | ENE | Rain | Cloudy | Fine |
| 5 | .05 | ● | 37 | 43 | 37 | .08 | .04 | 63 | 64 | ENE | ENE | Foggy | Fine | Cloudy |
| 6 | | | 41 | 45 | 39 | 29.94 | 29.87 | 64 | 65 | NE | E | Foggy | — | Fine |
| 7 | | | 40 | 44 | 38 | .84 | .87 | 65 | 65 | ENE | NE | — | Fine | Cloudy |
| 8 | | | 39 | 47 | 37 | .80 | .73 | 65 | 65 | NW | NW | Fine | Cloudy | — |
| 9 | | | 40 | 43 | 38 | .76 | .67 | 65 | 62 | NW | N | Fog | Fine | Fine |
| 10 | | | 39 | 45 | 33 | .72 | .74 | 62 | 62 | N | E | Fine | — | — |
| 11 | | | 38 | 42 | 34 | .70 | .68 | 62 | 60 | E | E | — | — | — |
| 12 | | ☽ | 37 | 41 | 35 | .62 | .56 | 60 | 60 | E | E | — | — | — |
| 13 | | | 39 | 43 | 35 | .51 | .64 | 60 | 65 | ENE | NE | — | — | Cloudy |
| 14 | | | 39 | 43 | 31 | .70 | .74 | 55 | 55 | NE | NNW | — | — | Fine |
| 15 | | | 34 | 42 | 28 | .71 | .69 | 54 | 54 | NE | NE | — | — | — |
| 16 | | | 36 | 40 | 29 | .64 | .51 | 54 | 53 | NE | ESE | Fog | — | — |
| 17 | | | 39 | 45 | 33 | .36 | .38 | 53 | 57 | ESE | SW | — | — | Cloudy |
| 18 | | | 45 | 54 | 45 | .58 | .60 | 57 | 53 | SW | S | — | — | Rain |
| 19 | .06 | | 57 | 59 | 51 | .51 | .36 | 52 | 55 | S | S | Fine | — | Fine |

The quantity of Rain fallen in the month of February, was 0 inch 26-100ths.

